# Optimization of Knitted Strain Sensor Structures for a Real-Time Korean Sign Language Translation Glove System

**DOI:** 10.3390/s25144270

**Published:** 2025-07-09

**Authors:** Youn-Hee Kim, You-Kyung Oh

**Affiliations:** Department of Convergence Design and Technology, Kookmin University, Seoul 02707, Republic of Korea; fbdk96@kookmin.ac.kr

**Keywords:** knitted strain sensors, plain plated stitch, smart glove, Korean Sign Language, textile integration system

## Abstract

Herein, an integrated system is developed based on knitted strain sensors for real-time translation of sign language into text and audio voices. To investigate how the structural characteristics of the knit affect the electrical performance, the position of the conductive yarn and the presence or absence of elastic yarn are set as experimental variables, and five distinct sensors are manufactured. A comprehensive analysis of the electrical and mechanical performance, including sensitivity, responsiveness, reliability, and repeatability, reveals that the sensor with a plain-plated-knit structure, no elastic yarn included, and the conductive yarn positioned uniformly on the back exhibits the best performance, with a gauge factor (GF) of 88. The sensor exhibited a response time of less than 0.1 s at 50 cycles per minute (cpm), demonstrating that it detects and responds promptly to finger joint bending movements. Moreover, it exhibits stable repeatability and reliability across various angles and speeds, confirming its optimization for sign language recognition applications. Based on this design, an integrated textile-based system is developed by incorporating the sensor, interconnections, snap connectors, and a microcontroller unit (MCU) with built-in Bluetooth Low Energy (BLE) technology into the knitted glove. The complete system successfully recognized 12 Korean Sign Language (KSL) gestures in real time and output them as both text and audio through a dedicated application, achieving a high recognition accuracy of 98.67%. Thus, the present study quantitatively elucidates the structure–performance relationship of a knitted sensor and proposes a wearable system that accounts for real-world usage environments, thereby demonstrating the commercialization potential of the technology.

## 1. Introduction

Humans use not only voice, but also facial expressions, body movements, and hand gestures to communicate accurately and effectively [1]. In particular, hand gestures have a wide variety of expressive forms, and have long been used as a means of communicating at the sentence level when opinions cannot be expressed through spoken language. Signed languages are conveyed by the hands, face, and body, and are primarily perceived visually [2]. However, without prior knowledge of sign language, it is difficult for non-signers to receive and understand this conversational communication. This creates a communication barrier between signers and non-signers [3]. To address this issue, there is a growing need for technologies that can recognize sign language and convert it into visual or auditory information.

Established techniques for capturing human finger movements include vision-based methods utilizing cameras and video, inertial measurement unit (IMU)-based approaches, and capacitive sensing methods. For instance, Yaseen et al. [4] implemented a vision-based real-time human–unmanned aerial vehicles (UAVs) interaction system in a virtual environment through a hybrid system for dynamic hand gesture recognition and reported stable classification accuracy. Zhang et al. [5] implemented a computer vision-based gesture interaction system for Building Information Modeling (BIM) and reported high recognition accuracy and stable real-time performance in various real-world environments. Park et al. [6] attached custom-developed IMU sensors to various body parts of construction workers—including the head, body, hands, and legs—and experimentally demonstrated that activities such as walking, jumping, standing, and working at heights could be recognized with approximately 90% accuracy. Pogrzeba et al. [7] recorded full-body kinematic data of daily activities from 32 participants across two age groups (young and old) using suits and gloves equipped with IMU sensors, thereby constructing a dataset that fills the gap in existing motion capture (MoCap) data. Atalay et al. [8] designed a highly stretchable textile–silicone capacitive sensor and demonstrated its feasibility for finger motion monitoring by integrating it into a glove using batch manufacturing technology. StretchSense Ltd. in New Zealand has developed a motion capture glove utilizing capacitive stretch fabric sensor technology to precisely track finger movements, demonstrating high accuracy and excellent responsiveness. While these technologies hold significant potential for monitoring finger movements, key challenges persist in wearable integration, scalable manufacturing, commercial viability, and cost-efficiency.

To improve upon these limitations, research on textile-based sensors is being actively conducted. Among them, knitted strain sensors are lightweight, flexible, and highly elastic, which makes them comfortable to wear for extended periods [9]. They can continuously monitor biosignals such as breathing and movement, thereby positioning them as key materials for human interface technologies. In particular, their excellent stretchability allows them to detect both small and large movements, which makes them well suited for monitoring finger joint motion. Based on these characteristics, the active development of knitted smart gloves for hand gesture recognition has been actively pursued in recent years. For instance, Ryu et al. [10] used a knitted glove sensor to analyze the electrical resistance behavior under compressive and tensile strains according to finger movements. The proposed sensor maintained excellent electrical properties and stability even after more than 400 repeated external deformations and five normal washing processes. Heo et al. [11] demonstrated a textile-based silver nanowire (AgNW) sensor with high conductivity (434.7 S/cm) on a polyester-based spandex substrate by utilizing stretchable and flexible polydimethylsiloxane (PDMS) films as both the interface/planarization and passivation layers. For hand gesture recognition, a full-sized glove-type sensor integrating five strain sensor units was designed, and it showed stable electrical responses to each finger movement. Lee et al. [12] fabricated sensors using the intarsia technique for hand posture pattern recognition and integrated them into a knitted glove. The proposed sensor exhibited a GF of 12 at a maximum strain of 10%, and classification of 10 hand postures from 10 subjects was achieved with an average accuracy of 94.17%. Similarly, Han et al. [13] used the intarsia technique to fabricate sensors with silver-plated nylon and nylon/spandex yarns. Considering the effects of elastic materials, sensor size, and glove size on performance, the optimal sensor was integrated into the glove. Tests on nine numeric gestures demonstrated that the proposed glove system provided stable recognition performance. Further, Qin et al. [14] developed a fabric sensor based on Ecoflex/carbon composite ink, which exhibited a high linear resistance change rate (R^2^ = 0.9965) over a strain range of 0–100%. In addition, it maintained excellent durability even after 2000 cycles of repeated stretching. This intelligent fabric sensor demonstrated the potential for human–computer interaction by monitoring finger movements. Cheng et al. [15] developed a flexible strain sensor using piezoresistive knitted fabrics and polydimethylsiloxane (PDMS), and evaluated its performance by attaching it to various body parts, including the fingers, wrist, upper arm, and thigh. The sensor demonstrated reliable detection of human movements, with a strain range of 0–87%, a GF between 3.7 and 10.6, stable responsiveness, and excellent durability.

While the abovementioned studies have demonstrated the capability of textile-based sensors to detect finger movements and their implementation in smart glove systems, the effects of various knitted structures on the electrical performance of these sensors have not been thoroughly investigated. Knitted fabrics have a structural mechanism in which the shape of the loops changes during the stretching and recovery processes, thus causing variations in the contact points between yarns [16]. This directly affects key sensor performance metrics such as sensitivity, responsiveness, reliability, and repeatability. Therefore, prior to integrating sensors into smart gloves, performance optimization based on knitted structural characteristics is essential for accurately detecting finger joint movements. This requires a comprehensive investigation of how factors such as knitting techniques, knit structures, and material combinations influence the electrical properties of the sensors. Moreover, studies on system-level integration for real-world applications remain limited. The entire process from sensor design to signal interface and final output must be organically configured, and a design that reflects the actual use environment is required. Accordingly, the objectives of this study are the following: (1) conduct an in-depth analysis of how the structural characteristics of knitted sensors affect their electrical performance; (2) design and fabricate high-performance sensors optimized for finger joint motion detection; and (3) develop a fully integrated textile-based system suitable for practical implementation in smart gloves.

In a previous study [17], the sensing characteristics of knitted strain sensors were analyzed based on knitting parameters such as stitch pattern, needle position (NP) number, and number of yarns. It was found that the plain stitch pattern, NP 12, and single-strand configuration were most effective for detecting subtle movements. Building on these findings, the present study maintains the same knitting conditions and conducts a detailed comparison of sensor performance based on two variables: the placement of the conductive yarn, and the presence or absence of elastic yarn. Five distinct sensor types were fabricated and subjected to 1000 bending cycles, with performance evaluated across different angles and speeds. Key performance indicators—including sensitivity, responsiveness, reliability, and repeatability—were used to identify the optimal configuration for recognizing finger joint movements. The selected sensor was then integrated into a glove to evaluate its real-time recognition of 12 Korean Sign Language (KSL) consonant and vowel gestures. Additionally, a mobile application was developed to convert recognized gestures into both text and voice output, thereby demonstrating the practical feasibility and scalability of the proposed sign language recognition system.

## 2. Materials and Methods

### 2.1. Knit Structure and Sensing Mechanism

Knitted fabric is formed by vertically interconnecting loops made from a single continuous yarn and is produced using various loop-based knitting techniques [18]. Structurally, knitted fabric consists of vertical wales and horizontal courses [19], with each loop comprising three segments: the head, the legs, and the sinker [20]. Importantly, Holm-type electrical contacts are generated when the head of one loop comes into contact with the sinker of an adjacent loop. When the knit is stretched in the wale direction, the contact surface and pressure between the head and sinker increase, the gap between loops narrows, and the number of contact points increases. As a result, a negative piezoresistive effect occurs, thereby lowering the resistance during stretching [21].

Based on this mechanism, the present study examines the electrical resistance behavior of knitted strain sensors under mechanical deformation. Two experimental variables are considered: (1) the placement of the conductive yarn, and (2) the inclusion of elastic yarn. To assess the effect of conductive yarn placement on loop contact points and conductive pathway formation, plain-plated-knit and plain-assembled-knit structures are compared. Additionally, the role of elastic yarn in modulating structural contact and pressure is analyzed by comparing two material configurations, including normal yarn–conductive yarn, and elastic yarn–conductive yarn combinations.

### 2.2. Knitted Sensor Fabrication

The knitted strain sensors were designed by adjusting variables such as sensor size, NP number, and loop location using the M1 Plus 7.2.037 pattern software (STOLL, Reutlingen, Germany). The sensors were then fabricated using a CMS330 KI W TT SPORT E7.2 (14-gauge) computerized flat knitting machine (STOLL, Reutlingen, Germany). The plain-plated-knit and plain-assembled-knit structures were then fabricated based on different knitting techniques [22]. Specifically, the plain plated knit is composed of two types of yarn and is characterized by loops arranged in parallel on the front and back sides. In the present study, this structure was made by combining non-conductive and conductive yarns. To ensure uniform distribution of the conductive yarn on either the front or back side of the fabric, the intarsia yarn carrier and plating yarn carrier were used separately. This method enabled consistent placement of the conductive yarn on a specific surface of the sensor. Meanwhile, the plain assembled knit is a structure in which two or more yarns—here, conductive and non-conductive yarns—are combined into a single loop using a single intarsia yarn carrier. Due to the twisting interaction between yarns during the knitting process, the conductive yarn was randomly distributed on both the front and back sides of the fabric. This led to a non-uniform and irregular distribution of the conductive material throughout the sensor.

The five fabricated sensor samples, produced using two different knitting techniques, are summarized in Table 1. The samples were produced with the plated (Pt) structure or with the assembled (As) structure, wherein the conductive yarn was distributed on either the back (b) or front (f) sides. For the As samples, where the conductive yarn distribution is inherently random due to the simultaneous feeding of yarns, the designation of front or back was determined based on the surface exhibiting a higher concentration of conductive yarn. Additionally, to investigate the effect of elasticity, two types of non-conductive yarns were used. In four of the samples, a non-elastic insulating layer was applied, consisting of a single strand of 2-ply blended yarn made from acrylic (A) and wool (W) in a 1:1 ratio, supplied by C&TEX (Seoul, Republic of Korea). In contrast, one sample included an elastic insulating layer, composed of two strands of 3-ply blended yarn containing rayon (R), nylon (N), polyester (P), and wool (W) in a 42:20:10:28 ratio, along with a single strand of spandex (SPAN) yarn, supplied by C&TEX (Seoul, Republic of Korea). All five samples had the same overall dimensions of 85 mm × 260 mm. The sensor area was designed to be 20 mm × 40 mm, which was considered an optimal balance for effectively detecting localized tensile strain during motion while minimizing the influence of unintended surrounding movements. The conductive yarn used in each sensor was a polyamide (PA)–polyester (P) blend (<530 Ω/m) manufactured by AMANN (Bönnigheim, Germany).

### 2.3. Fabrication of the KSL Glove

In this study, a smart glove was fabricated as an interface device to facilitate communication between sign language users and non-users. The main body of the glove was knitted using two strands of A/W regular yarn. For improved wearability, the dorsal and palm areas of the hand were constructed using a plain-knit structure, while the wrist area was knitted in a 2 × 1 rib structure. Furthermore, the glove was custom-fitted to the user’s hand size to ensure accurate stretching during finger flexion. A 20 mm × 40 mm knitted strain sensor was fabricated by combining conductive yarn (<530 Ω/m) with the insulating A/W yarn. The placement of the knitted strain sensors on the glove was determined based on anatomical landmarks and empirical observations of finger joint movement. Sensors were positioned on the dorsal side of the fingers, directly above the proximal interphalangeal (PIP) and metacarpophalangeal (MCP) joints, where the greatest strain occurs during flexion and initial bending typically begins [10]. To optimize sensor positioning, preliminary bending tests were conducted to identify regions exhibiting the highest and most consistent strain responses during representative finger gestures. Sensor alignment was then refined to maximize sensitivity to motion while minimizing signal interference from adjacent joints. The interconnection lines were stitched using conductive yarn (<85 Ω/m) manufactured by AMANN (Bönnigheim, Germany) in a zigzag pattern with approximately 3 mm spacing. To independently detect the motion of each finger, two signal lines were added per finger. These lines started from the upper and lower edges of each sensor, at an internal offset of 5 mm, and extended to the ground (GND) terminal area. A total of 10 snap connectors (5 mm in diameter) were attached to the ends of the signal lines to interface with the hardware.

### 2.4. Experimental Setup for Performance Evaluation

As detailed in the following paragraphs, dynamic bending tests were initially performed in order to optimize the knitted strain sensors for response to finger movements, after which the optimized sensor was integrated into a glove and its motion recognition performance was evaluated by user testing.

#### 2.4.1. Repetitive Bending Test

The knitted strain sensor used in this study is inherently designed to detect tensile strain rather than bending itself. However, by attaching the sensor along the dorsal side of the finger joints, bending-induced elongation on the outer surface of the finger was captured as measurable strain. This approach allows indirect estimation of finger bending based on the corresponding tensile deformation.

**Figure 1:**
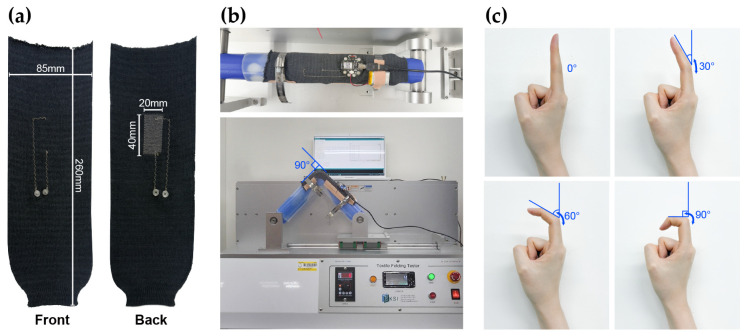
Experimental setup for the bending test: (**a**) front and back sides of the strain sensor; (**b**) E-textile folding tester; (**c**) applied bending angles of 30°, 60°, and 90°.

Dynamic bending tests were performed to verify whether the sensors could recognize complex finger movements and provide consistent measurements. The front and back sides of the sensors used in the experiment are presented in Figure 1a. The electrical characteristics of the sensors were evaluated using an E-textile flexing tester (CKFT-T400, Netest, Hwasung-si, Republic of Korea), as shown in Figure 1b. Prior to testing, each sample was subjected to 5 pre-bending cycles to ensure stable and uniform electrical signals [23]. The dynamic bending tests were performed for 1000 cycles each, with bending angles of 30°, 60°, and 90°, at speeds of 10, 30, and 50 cycles per minute (cpm). These bending angles were chosen to simulate actual finger movements, and the corresponding finger motions for each angle are shown in Figure 1c.

For electrical connectivity to the hardware, a lock stitch was employed for the signal line, and a snap fastener was used as the GND terminal. The interconnection line was fabricated using silver-coated PA/P conductive yarn, which exhibits a relatively low resistance of less than 85 Ω/m. Taking the fabric’s elasticity into account, the interconnection lines were sewn in a zigzag pattern with an approximate spacing of 3 mm to maintain flexibility. Two interconnection lines were routed from points 5 mm inside the top and bottom edges of the sensor to the GND terminal. Snap connectors with a diameter of 5 mm were attached at the ends of these lines to facilitate coupling with the external hardware system.

Data acquisition from the sensor was performed using a microcontroller unit (MCU) based on Arduino (ESP32-PICO-V3, Indifrog, Seongnam-si, Republic of Korea). The reference voltage was set to 3.3 V, and a fixed resistance of 100 Ω was used. The MCU converted the analog voltage signal generated by sensor deformation into a digital value ranging from 0 to 4095 through an analog-to-digital converter (ADC). To determine an appropriate sampling rate, the dynamic characteristics of finger movement were considered. People perform cyclical flexion–extension movements at an average frequency of approximately 2 Hz per finger [24]. Lei et al. [25] reported that a sampling frequency of 5 Hz is sufficient for classifying static finger movements. According to the Nyquist theorem, to accurately digitize an analog signal, the sampling frequency must be at least twice the highest frequency component of the signal. Therefore, considering the maximum frequency of finger movements (approximately 5 Hz), this study sampled the strain sensor signals at 10 Hz. This sampling rate was chosen to avoid excessive data collection and to improve processing efficiency while maintaining precise differentiation of finger movements. During repeated bending and recovery motions, the electrical signals from the sensor were output to the serial monitor at 0.1 s intervals via an Arduino program. The collected data were analyzed to assess the sensitivity, responsiveness, reliability, and repeatability of the sensor.

#### 2.4.2. KSL Smart Glove Motion Recognition Test

The selected sensor was integrated into the KSL smart glove in order to evaluate the possibility of fingerspelling recognition via user tests. Fingerspelling is a method of expressing Korean consonants and vowels by using the shapes of the hand and fingers to supplement vocabulary that is difficult to express in sign language [26]. KSL fingerspelling consists of a total of 24 characters, made up of 14 consonants and 10 vowels [27]. In this study, experiments were conducted on 10 consonants (ㄱ, ㄹ, ㅁ, ㅂ, ㅅ, ㅇ, ㅈ, ㅊ, ㅋ, and ㅎ) and 2 vowels (ㅏ and ㅣ) that can be distinguished by fingerspelling using the knitted strain sensor.

To verify the smart glove’s capability for real-time KSL gesture recognition, a smartphone application was developed. Data were collected from a single participant who maintained the initial state (no finger bending) for 3 s and the gesture state (finger bending) for 6 s at a consistent pace, repeating this cycle 25 times. The smart glove’s Bluetooth module was paired one-to-one with the Bluetooth Terminal app, reliably receiving sensor signals at 1 s intervals, and the finger movement information was displayed in real time as text and voice on a mobile device.

The experiments were conducted in a typical indoor environment (approx. 23 ± 1 °C) within a university electronics lab, where a standard Wi-Fi router was in operation and no electromagnetic shielding was employed. While no explicit electromagnetic disturbances were introduced, the test conditions reflect a general urban usage scenario.

To ensure accurate signal acquisition and minimize external noise interference, the analog voltage signals generated by the knitted strain sensors were digitized using the differential input mode of the MCU’s ADC. This configuration allows the ADC to measure only the voltage difference between two input lines, effectively rejecting common-mode noise such as electromagnetic interference (EMI) from nearby electronic devices. As a result, the sensor readings maintained high reliability, even under typical urban indoor conditions where Wi-Fi routers and other sources of electromagnetic radiation were present.

## 3. Results and Discussion

### 3.1. Deformation Mechanism of Five Types of Knitted Strain Sensors

**Figure 2:**
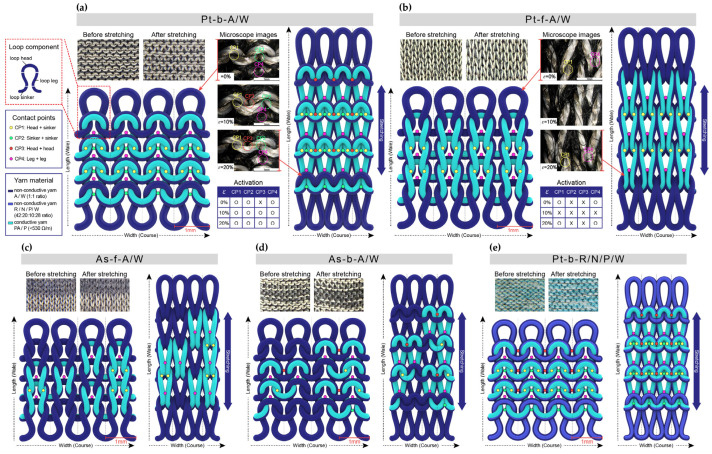
An analysis of the deformation mechanisms in terms of the formation of contact points before and after stretching for (**a**) Pt-b-A/W; (**b**) Pt-f-A/W; (**c**) As-f-A/W; (**d**) As-b-A/W; and (**e**) Pt-b-R/N/P/W.

The structural changes in the contact points formed between the conductive yarns during stretching and recovery for each of the five distinct sensors are revealed by the 3D modeling images in Figure 2. When bent, the sensors are stretched along the wale direction, which changes the contact positions between the conductive yarns [28]. These contact points can be classified into interlocking contacts, where physical contact occurs, and jamming contacts, where contact does not occur. The various contact points are labeled as follows: yellow (CP1) indicates contact between the loop head and sinker loop, green (CP2) indicates contact between sinker loops, red (CP3) indicates contact between loop heads, and pink (CP4) indicates contact between loop legs.

The changes in contact points before and after stretching for Pt-b-A/W are shown in Figure 2a. Before stretching, interlocking contacts (CP1, CP2, and CP4) and a jamming contact (CP3) are seen to coexist. After 10% strain, however, the CP1, CP2, CP3, and CP4 contacts have all transitioned into interlocking contacts. By contrast, the Pt-f-A/W exhibits interlocking contacts (CP1 and CP4) and jamming contacts (CP2 and CP3) before stretching, as shown in Figure 2b. Moreover, while all of these contacts have switched to jamming contacts at 10% strain, they are seen to have reverted to interlocking contacts and jamming contacts at 20% strain. The same contact point changes are observed for As-f-A/W, as shown in Figure 2c. However, because the conductive yarns are randomly arranged front and back, the number of contact points is relatively lower than Pt-f-A/W. Meanwhile, As-b-A/W exhibits the same contact point changes as Pt-b-A/W, as shown in Figure 2d, but with a reduced number of contact points due to the irregular arrangement of yarns. Finally, Pt-b-R/N/P/W has the same structure as Pt-b-A/W but exhibits different contact point changes due to the use of elastic yarns, as shown in Figure 2e. The high elasticity causes high pressure to be applied between loops before stretching, and structural deformation progresses slowly during stretching, thus resulting in minimal changes in the number of contact points. This structural analysis helps to understand the differences in sensing mechanisms depending on the arrangement of conductive yarns and the presence or absence of elastic yarns, thus providing foundational data for the electrical characterization.

### 3.2. Dynamic Bending Test Results

#### 3.2.1. The Bending Test Results and Initial Analysis for All Five Samples

**Figure 3:**
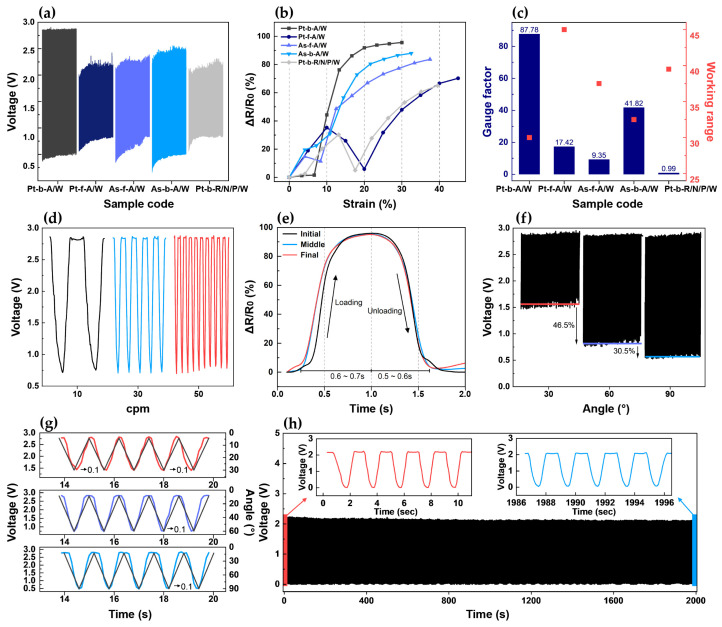
Dynamic bending test results for the five samples, along with an in-depth analysis of the results for Pt-b-A/W: (**a**) voltage outputs of the five samples during 1000 bending cycles at 90°; (**b**) relative resistance changes of the five samples at 90° and 30 cpm; (**c**) the corresponding GF values and working ranges; (**d**) voltage fluctuations of Pt-b-A/W according to bending speed at 90°; (**e**) loading and unloading times at the beginning, middle, and end of 1000 cycles at 90° and 30 cpm; (**f**) voltage outputs during 1000 cycles of bending to angles of 30°, 60°, and 90° at 50 cpm; (**g**) voltage change at angles of 30°, 60°, and 90° at 50 rpm; and (**h**) voltage changes during 1000 cycles at 90° and 30 cpm.

The dynamic bending test results for all five samples are presented in Figure 3a. Here, Pt-b-A/W showed the largest voltage change, thereby demonstrating that the knit structure was a key factor influencing the output voltage of the strain sensor. Based on the bending test data, the corresponding strain data at 30 cpm are presented in Figure 3b. The resistance of the knitted strain sensor was calculated indirectly by measuring the voltage drop across a known reference resistor in a voltage divider configuration. Specifically, the voltage signal was converted to resistance using Equation (1), where $R_{sensor}$ is the resistance of sensor, $V_{ref}$ is the supply voltage (3.3 V), $V_{out}$ is the output voltage measured from the sensor, and $R_{ref}$ is the reference resistance (100 Ω).

(1) $$R_{sensor}=\frac{V_{out}}{(V_{ref}-V_{out})/R_{ref}}$$

Here, one cycle was extracted from the same section of each sample to analyze the resistance change pattern according to strain. Notably, Pt-b-A/W showed the largest resistance change rate, with a linear increase in resistance as the strain increased from 7% to 30%. Meanwhile, Pt-f-A/W showed an initial increase in resistance as the strain was increased up to 10%, followed by a decrease until 20% strain, and then an increase again at strains of up to 45%. Similarly, As-f-A/W showed an initial increase in resistance at strains of up to 5%, followed by a decrease until 10% strain, and then another increase at strains of up to 37.5%. By contrast, As-b-A/W showed a sharp increase in resistance at strains of up to 4%, followed by a more gradual increase at strains of up to 10%, after which the resistance continued to increase even more rapidly at strains of up to 32.5%, thus resulting in the second-highest resistance change after Pt-b-A/W. Finally, Pt-b-R/N/P/W showed an increase in resistance at strains of up to 13%, followed by a decrease at strains of up to 18%, and then another increase at strains of up to 39.5%. Thus, in brief, Pt-f-A/W, As-f-A/W, and Pt-b-R/N/P/W each exhibited nonlinear behavior, with the resistance values both increasing and decreasing with the increase in strain, rather than exhibiting a consistent increase. Meanwhile, As-b-A/W showed a continual increase, but with a variable rate of change, while Pt-b-A/W showed a large resistance change along with a linear response to strain. These results were consistent with the abovementioned contact point changes during structural deformation. Pt-b-A/W, which had the greatest and most steadily increasing number of contact points, showed the best sensor performance, while the other samples each exhibited irregular increases in contact points and relatively unstable resistance changes. The GF value of each sample was calculated by using Equation (2):

(2) $$GF=\frac{\frac{∆R}{R_{0}}}{\frac{∆L}{L_{0}}}=\frac{∆R}{\varepsilon R_{0}}$$

where $R_{0}$ is the min resistance, ∆R is the applied resistance, $L_{0}$ is the initial length, ∆L is the applied length, and ε is the strain value. The results are shown in Figure 3c. Because the knitted strain sensors used herein each exhibited a negative piezoresistive effect, whereby the resistance decreased with increased stretching, the GF was calculated by taking the initial resistance value as the maximum and the resistance after stretching as the minimum [29]. To measure small movements such as those of the fingers, sensors with high sensitivity even within a low working range are required. In this respect, although Pt-b-A/W showed the narrowest working range among the five samples, it had a GF of up to ~89 times that of the other samples. These results clearly demonstrate that differences in the knit structure significantly affect the sensor’s electrical performance, including its sensitivity, working range, and linearity of resistance change. Accordingly, a further in-depth analysis was conducted in order to determine whether the best-performing sample (i.e., Pt-b-A/W) was suitable for monitoring complex and continuous finger bending motions.

#### 3.2.2. In-Depth Analysis of the Bending Test Results for Pt-b-A/W

The voltage changes of Pt-b-A/W at a fixed bending angle of 90° under various bending speeds of 10, 30, and 50 cpm are shown in Figure 3d. Here, the output signals were found to be stable across the various bending speeds. This indicates that the sensor can reliably detect finger movements at various speeds with consistent performance. Further, the voltage responses of Pt-b-A/W during the initial, middle, and final stages of bending to 90° at 30 cpm for 1000 cycles (with one cycle extracted from each stage) are shown in Figure 3e. This enables observation of the change in sensor responsiveness over time. Specifically, the sensor’s response speed was evaluated by measuring the loading (bending) and unloading (recovery) times during each cycle. At 30 cpm, one cycle takes an average of 2 s. Thus, during the initial stage, Pt-b-A/W showed a loading time of 0.6 s and an unloading time of 0.5 s. In the middle and final stages, the loading time was 0.7 s, and the unloading time was 0.6 s. Although the loading and unloading times showed a difference of 0.1 s each compared to the initial stage, they remained consistent throughout the middle and final stages, thereby confirming that the operation was completed within 2 s. In other words, despite a slight delay compared to the initial stage, the response stabilizes as the cycles repeat, thereby suggesting that the sensor can measure repetitive movements in real time. The voltage responses of Pt-b-A/W during 1000 cycles of dynamic bending to angles of 30°, 60°, and 90° at a fixed speed of 50 cpm are shown in Figure 3f. Thus, at 30° bending (red line), the average minimum voltage was 46.5% higher than that at 60° bending (purple line), while the average minimum voltage at 60° bending was 30.5% higher than that at 90° bending (blue line). These changes in voltage according to the bending angle demonstrate that the sensor is able to provide distinct signals in response to joint movements. This suggests that the sensor can differentiate between various finger bending angles, thus making it applicable for recognizing sign language motions. The responsiveness of Pt-b-A/W at 50 cpm was further evaluated by overlaying the voltage values at 30°, 60°, and 90° on the graph of bending angle (black line) in Figure 3g. Here, five cycles were extracted from the same section for each bending angle. Notably, the voltage curves were seen to match the bending angle curve closely, thereby confirming the excellent responsiveness of the strain sensor to changes in angle. Moreover, although a response delay of about 0.1 s was observed for all three angle conditions, this did not lead to significant performance degradation in practical application, as the maximum speed of typical human hand movements is approximately 1 m/s [30]. Therefore, the sensor is able to measure joint motions in real time. The changes in output voltage of Pt-b-A/W during 1000 bending cycles to an angle of 90° at a fixed speed of 30 cpm are presented in Figure 3h. Here, the voltage showed a stable response without significant changes between the first and last five cycles, thereby demonstrating the excellent repeatability and durability of the sensor. Taken together, the above results indicate that the sensor with the plated structure provides excellent performance in terms of sensitivity, responsiveness, reliability, and repeatability. This is attributed to the effectiveness of the contact–separation mechanism when the conductive yarn is evenly distributed on the back side of the glove. Additionally, the absence of elastic yarn allows structural deformation to occur rapidly, thereby inducing numerous changes in contact points. Hence, the following section examines the practical application of a smart glove based on Pt-b-A/W for the recognition of KSL.

### 3.3. Development and Performance Evaluation of a KSL Smart Glove Translation System

#### 3.3.1. Development

**Figure 4:**
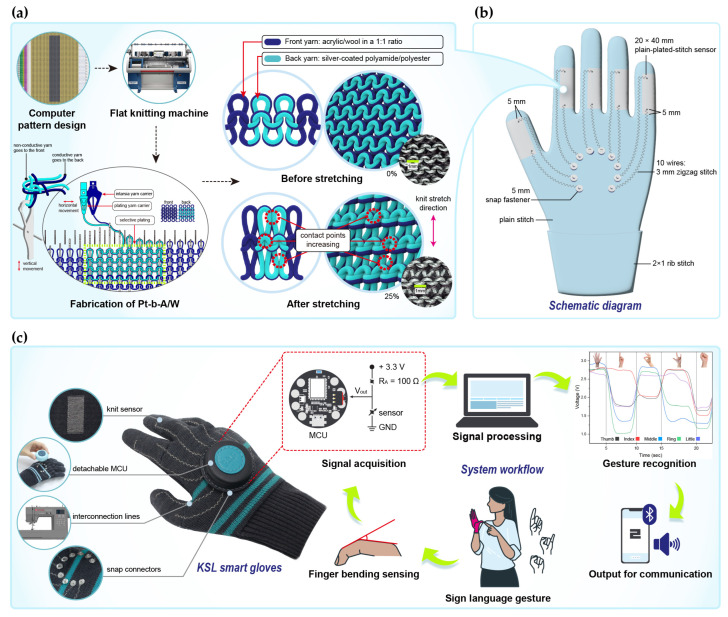
The fabrication, integration, and implementation of a smart glove for KSL gesture recognition: (**a**) fabrication of the plain-plated-stitch sensor and sensing mechanism; (**b**) a schematic diagram of the smart glove integrating the sensor, interconnection lines, and snap buttons; (**c**) photographic image showing the process of recognizing KSL gestures using the as-developed smart glove.

The design process and testing of the textile integration system for recognizing KSL gestures are shown in Figure 4. As shown in Figure 4a, the sensor was fabricated using a plain-plated-knit structure in which the conductive yarn is positioned on the back side of the fabric. The 3D structural representation on the right demonstrates how this configuration enables uniform loop deformation during stretching, thereby ensuring stable contact between conductive yarns.

Figure 4b depicts the wearable system integrating the knitted sensors, interconnection lines, and snap connectors. Sensors were placed between the PIP and MCP joints to detect finger movements independently, with two signal lines per finger totaling ten wires. These lines extend from slightly above and below each sensor toward the back of the hand. The circular snap buttons (5 mm diameter) attached at the wire ends are located over the meta-carpal bones to reduce movement discomfort.

A photographic image of the as-fabricated KSL recognition smart glove is presented in Figure 4c. The glove integrates four components: the knitted sensor, interconnection lines, snap connectors, and MCU. The knitted sensor is positioned on the inside surface of the glove to protect it from external contamination and physical damage. As the sensor is not externally exposed, the glove can be worn naturally during daily activities without compromising appearance or comfort. The interconnection lines are fabricated with a zigzag stitch using a sewing machine and are integrated with the glove, thus providing excellent wearing comfort without any sense of incongruity. The snap interconnector allows for easy attachment and detachment of the device to facilitate washing and maintenance of the gloves. The MCU is enclosed in a leather casing to protect it from external contaminants and scratches, while also enhancing the glove’s aesthetic appeal and user-friendliness. Overall, the textile integration system was designed with key practical considerations in mind, including sensor protection, wearing comfort, ease of maintenance, and usability. Additionally, the straightforward fabrication process supports scalability and mass production. The process of recognizing KSL gestures using the developed smart glove is as follows: (1) While wearing the glove, the user performs sign language gestures by bending their fingers. (2) The degree of finger bending is detected by the integrated strain sensors.(3) The signals are processed by the MCU and interpreted as specific consonants or vowels. (4) The processed data are transmitted in real time to a smartphone via a BLE wireless communication module. (5) The mobile application converts the received data into both voice and text to provide intuitive feedback to the user. The performance of this KSL smart glove motion recognition system is evaluated in the following section.

#### 3.3.2. Real-Time Performance Evaluation of the Smart Glove for KSL Fingerspelling

**Figure 5:**
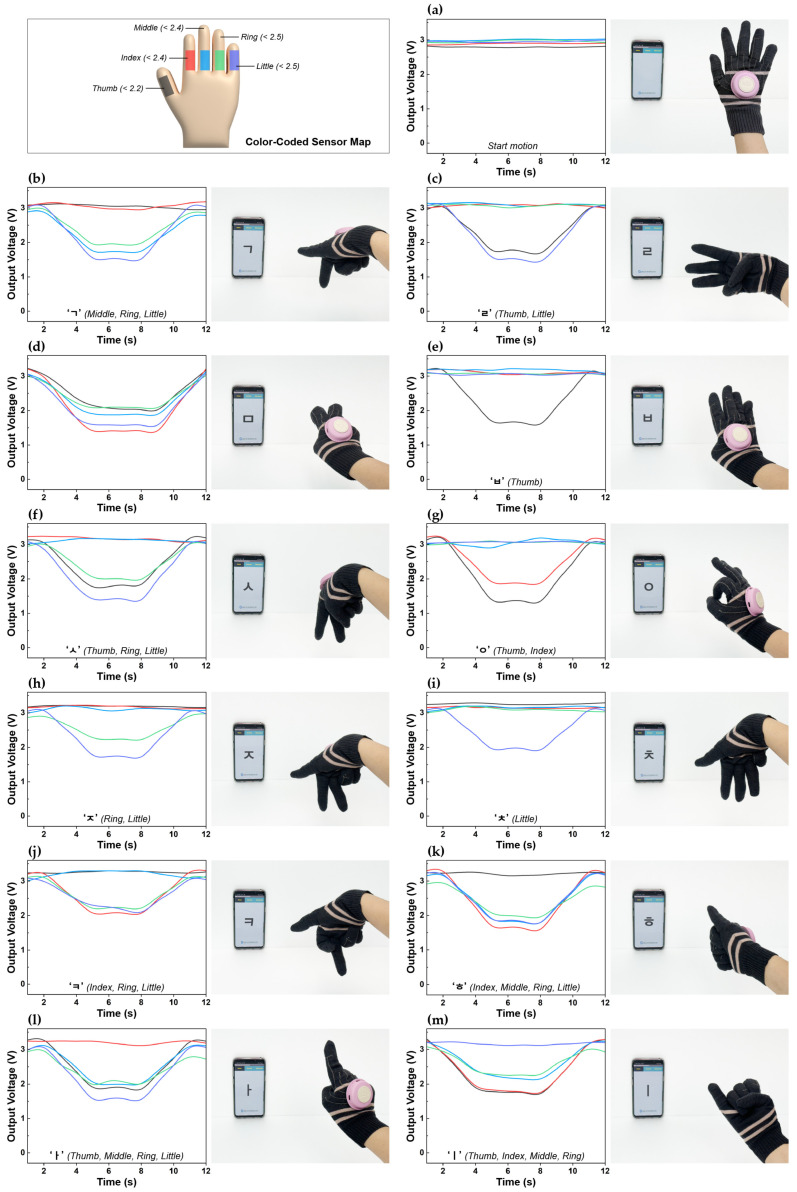
A demonstration of the knitted glove sensing system for the recognition of KSL hand motions using a wireless system: (**a**) start position; (**b**) motion ‘ㄱ’; (**c**) motion ‘ㄹ’; (**d**) motion ‘ㅁ’; (**e**) motion ‘ㅂ’; (**f**) motion ‘ㅅ’; (**g**) motion ‘ㅇ’; (**h**) motion ‘ㅈ’; (**i**) motion ‘ㅊ’; (**j**) motion ‘ㅋ’; (**k**) motion ‘ㅎ’; (**l**) motion ‘ㅏ’; and (**m**) motion ‘ㅣ’; Color-Coded Sensor Map: black (thumb), red (index), blue (middle), green (ring), and purple (little).

The voltage output signals obtained when performing fingerspelling motions while wearing the glove are shown in Figure 5, along with images displaying the corresponding real-time text and voice translations that are output through the mobile application. The colored lines in the graph represent different fingers: thumb (black), index (red), middle (blue), ring (green), and little (purple).

In this study, we implemented a simple threshold-based algorithm to distinguish various finger movements. Voltage amplitude ranges for each gesture were pre-determined through empirical testing, and decision logic was applied accordingly. Specifically, voltage thresholds were set for each finger based on their respective minimum bending voltages (thumb: 2.2 V; index and middle fingers: 2.4 V; ring and little fingers: 2.5 V). Using these criteria, gestures were systematically identified according to the combinations of fingers involved.

To ensure fast response and enable real-time processing, the raw voltage signals were processed using the Adjacent-Averaging filter provided by OriginLab. Based on these processed signals, all 12 fingerspelling gestures were successfully classified, and the sensor exhibited high sensitivity to subtle differences in bending angles.

For example, although the consonants ‘ㄹ’ (thumb + little finger) and ‘ㅇ’ (thumb + index finger) have similar structures, a greater voltage change was observed for ‘ㅇ’ (3.2–1.4 V) due to its larger thumb bending angle, compared to ‘ㄹ’ (3.1–1.7 V). This indicates that the sensor is capable of distinguishing fine variations in movement based on voltage output. However, in some gestures, such as ‘ㅁ’, ‘ㅅ’, ‘ㅏ’, and ‘ㅣ’, a larger bending angle did not necessarily result in lower voltage values. This is likely due to differences in the deformation applied to the sensor depending on the combination of fingers being bent. In other words, even when the same finger is flexed, the direction, magnitude, and distribution of strain applied to the sensor can vary depending on which other fingers move together. Accordingly, both the voltage threshold of each finger and the interplay of multiple finger movements were taken into account to enhance classification accuracy.

Before finger movement, the baseline output voltage ranges from 2.75 to 3.04 V (Figure 5a). When specific fingers are bent to represent Korean consonants and vowels, distinct voltage patterns are observed. For instance, during the ‘ᄀ’ motion involving the middle, ring, and little fingers, the voltage drops to 1.71, 1.89, and 1.48 V, respectively (Figure 5b). During the ‘ㄹ’ motion, which involves the thumb and little fingers, the voltage drops to 1.66 and 1.44 V, respectively (Figure 5c). For the ‘ㅁ’ motion, which involves all five fingers, the voltage drops to 2.02, 1.39, 1.88, 2.06 and 1.57 V, respectively (Figure 5d). During the ‘ㅂ’ motion, which involves only the thumb, the voltage drops to 1.6 V (Figure 5e). For the ‘ㅅ’ motion, which involves the thumb, ring finger, and little finger, the voltage drops to 1.74, 1.99 and 1.39 V, respectively (Figure 5f). The ‘ㅇ’ motion, which involves the thumb and index fingers, causes the voltage to drop to 1.36 and 1.85 V, respectively (Figure 5g). For the ‘ㅈ’ motion, which involves the ring and little fingers, the voltage drops to 2.2 and 1.72 V, respectively (Figure 5h). During the ‘ㅊ’ motion, which involves only the little finger, the voltage drops to 1.87 V (Figure 5i). For the ‘ㅋ’ motion, which involves the index, ring, and little fingers, the voltage drops to 2.05, 2.16, and 2.06 V, respectively (Figure 5j). The ‘ㅎ’ motion, which involves the index, middle, ring, and little fingers, causes the voltage to drop to 1.58, 1.78, 1.95, and 1.79 V, respectively (Figure 5k). When performing the ‘ㅏ’ motion, which involves the thumb, middle, ring, and little fingers, the voltage drops to 1.82, 1.95, 1.79, and 1.54 V, respectively (Figure 5l). Finally, the ‘l’ motion, which involves the thumb, index, middle and ring fingers, causes the voltage to drop to 1.71, 1.75, 2.11, and 2.25 V, respectively (Figure 5m).

To evaluate the classification accuracy of each gesture, data were collected by repeating each gesture 25 times, and the results are presented in the confusion matrix shown in Table 2. Among the 12 gestures, 9 achieved a recognition rate of 100%, while ‘ㅁ’ and ‘ㅋ’ recorded recognition rates of 96%, and ‘ㅣ’ achieved 92%. Misclassifications for ‘ㅁ’ and ‘ㅣ’ were mainly due to incomplete detection of the middle finger, and the lower accuracy for ‘ㅋ’ was attributed to improper recognition of the little finger. This is likely caused by slight bending differences that led to voltage values exceeding the threshold. Despite these errors, the overall average recognition rate was 98.67%, demonstrating excellent performance.

These consistent and simultaneous voltage responses confirm the sensor’s high reliability and rapid response during fingerspelling motions. In other words, these results demonstrate the structural excellence of the Pt-b-A/W sample and also confirm the practical feasibility of the textile integration system implemented using this fabric.

## 4. Conclusions

This study investigated the relationship between the structural deformation mechanism and the electrical performance of knitted strain sensors, focusing on two key design variables: the placement of conductive yarn, and the inclusion of elastic yarn. Among the five configurations evaluated, the plated structure with conductive yarn uniformly positioned on the back side and without elastic yarn (designated as Pt-b-A/W) demonstrated the most optimal performance for finger bending detection. This structure promoted uniform loop deformation and rapid structural responsiveness, resulting in superior sensor characteristics, including a gauge factor (GF) up to 89 times greater than those of other configurations. The sensor exhibited a response time of less than 0.1 s at 50 cycles per minute (cpm), while maintaining high performance across various bending angles and speeds.

Based on this optimized sensor, a smart glove system for Korean Sign Language (KSL) recognition was developed. The smart glove integrated the optimized knitted strain sensor with interconnection lines, snap connectors, and a microcontroller unit (MCU) to achieve excellent wearability, easy maintenance, and user convenience. The glove successfully recognized 12 KSL fingerspelling gestures in real time and translated them into both text and audio outputs via a smartphone application, thereby validating its practical feasibility. The overall average recognition accuracy reached 98.67%, demonstrating excellent performance.

Unlike previous studies, this work provides an in-depth analysis of how knitted structural design directly influences sensor performance and extends these findings into a fully integrated wearable system. The high-performance sensor developed in this study can be applied to various parts of the body, making it promising for a wide range of applications such as healthcare, rehabilitation, and human–computer interaction. Moreover, the modular design of the textile-integrated system offers scalability and supports mass production, suggesting a viable commercialization path for next-generation wearable technologies.

## Figures and Tables

**Table 1:** Photographs and experimental characterization of the knitted strain sensor samples.

**Sample code**	Pt-b-A/W		Pt-f-A/W		As-f-A/W		As-b-A/W		Pt-b-R/N/P/W	
**Structure**	plated		plated		assembled		assembled		plated	
**Conductive yarn position**	back side		front side		mainly front side		mainly back side		back side	
**Photographic** **image**	front	back	front	back	front	back	front	back	front	back
	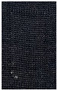	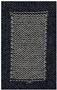	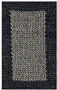	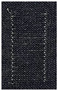	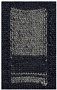	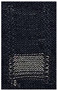	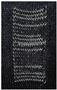	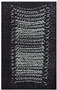	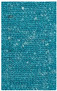	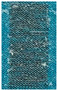
**Yarn** **composition**	2-ply A/W + conductive yarn								3-ply R/N/P/W + span + conductive yarn	

**Table 2:** The confusion matrix showing the classification accuracy (%) for the KSL hand gesture recognition.

KSL	ㄱ	ㄹ	ㅁ	ㅂ	ㅅ	ㅇ	ㅈ	ㅊ	ㅋ	ㅎ	ㅏ	ㅣ	Unrecognized	Accuracy (%)
ㄱ	25													100
ㄹ		25												100
ㅁ			24										1	96
ㅂ				25										100
ㅅ					25									100
ㅇ						25								100
ㅈ							25							100
ㅊ								25						100
ㅋ									24				1	96
ㅎ										25				100
ㅏ											25			100
ㅣ												23	2	92

## Data Availability

Data are contained within the article.
